# Managing High-Risk Root Caries with Silver Diamine Fluoride Application: A Markov Simulation Study

**DOI:** 10.36469/001c.160210

**Published:** 2026-05-14

**Authors:** Young S. Kim, Ji W. Yoo, Maryam Tabrizi, Jae-In Ryu, Yonsu Kim, Maria Teresa Chong, Dong-Hun Han, Jong Ho Won, Yoohwan Kim, Iulia Ioanitoaia-Chaudhry, Mark J. Rosenberg, Marcia Loo, Claire S. Lee, Anderson Penna

**Affiliations:** 1 Department of Anesthesiology and Pain Medicine Korea University Guro Hospital, Seoul, Republic of Korea; 2 Department of Internal Medicine Kirk Kerkorian School of Medicine at UNLV, Las Vegas, Nevada, USA; 3 Department of Biomedical Sciences UNLV School of Dental Medicine, Las Vegas, Nevada, USA; 4 Department of Preventive and Social Dentistry Kyung Hee University College of Dentistry, Seoul, Republic of Korea; 5 Department of Healthcare Administration and Policy School of Public Health, UNLV, Las Vegas, Nevada, USA; 6 Department of Family and Community Medicine Kirk Kerkorian School of Medicine at UNLV, Las Vegas, Nevada, USA; 7 Department of Preventive and Social Dentistry Seoul National University School of Dentistry, Seoul, Republic of Korea; 8 Department of Urban Design and Planning Hongik University College of Engineering, Seoul, Republic of Korea; 9 Department of Computer Science Howard Hughes College of Engineering, UNLV, Las Vegas, Nevada, USA; 10 Geriatric Education Center VA Southern Nevada Healthcare System, North Las Vegas, Nevada, USA; 11 Tonopah Dental Center, Tonopah, Nevada, USA; 12 Nevada Dental Foundation, Carson City, Nevada, USA; 13 Department of Clinical Sciences UNLV School of Dental Medicine, Las Vegas, Nevada, USA; 14 Emory University, Atlanta, Georgia, USA; 15 The Connection Sphere, Las Vegas, Nevada, USA

**Keywords:** Medicaid, caries, Markov model, orosystemic, silver

## Abstract

**Background:**

Dental caries are common oral health conditions among Medicaid beneficiaries. Silver diamine fluoride (SDF) has been applied to manage dental caries.

**Objectives:**

To evaluate the cost-effectiveness of SDF application for managing high-risk root caries within the context of Medicaid, a Markov simulation model was adopted from a healthcare system perspective.

**Methods:**

A 45-year-old subject with 21 residual teeth was simulated for 19 years. Twice-yearly 38% SDF application on the tooth surface at risk for caries was compared with 5% sodium fluoride (NaF) varnish in terms of effectiveness and cost. Tooth-years free of extraction were set as the effect.

**Results:**

Compared with 5% NaF varnish, 38% SDF application was cost-effective, with a difference of 6.34 extraction-free tooth-years and an additional cost of 7798(incrementalcost−of−effectivenessratio,1229 per extraction-free tooth-year gained; incremental net monetary benefit, $309 355). One-way sensitivity analyses on SDF and NaF costs confirmed cost-effectiveness of SDF application over NaF varnish.

**Conclusions:**

An integrated orosystemic care model is underscored to disseminate the benefits of SDF application as the Medicaid payment system shifts from fee-for-service to managed care. Semiannual 38% SDF application might reduce and delay tooth extraction due to root caries in statewide dental provider shortage areas, particularly “dental deserts,” where Medicaid-accepting dentists are not available.

## INTRODUCTION

Dental caries is the demineralization and cavitation due to bacteria and is a common oral health condition in the United States (US).^1,2^ The root may be exposed because of gingival recession of previous periodontal disease; this exposed root surface is softer than the enamel-covered crown of the tooth.^1,2^ Since root caries are often difficult to clean and generally asymptomatic, they may develop into an advanced stage before discovery.^1,2^ The risk factors of root caries are poor oral hygiene associated with comorbidities, such as diabetes mellitus and substance use disorders, as well as limited access to dental care among individuals living with disabilities, such as Medicaid beneficiaries.^1^ Silver diamine fluoride (SDF) 38%, a brush-on therapy, has an antimicrobial effect on cariogenic biofilms and can be applied to arrest and prevent dental caries, except irreversible pulpitis.^1,3^ Sodium fluoride (NaF) varnish has been proven to prevent root caries by reducing the solubility of dental tissue and inhibiting cariogenic bacterial activity.^1,3^ It is presumed that 38% SDF application to manage root caries is more potent than NaF varnish.^1,3^ Multiple applications over years are recommended to sustain effectiveness of 38% SDF application to manage root caries, particularly for those with high-risk root caries.^1,3^

Although 38% SDF has been increasingly applied in clinical practice, evidence on its economic value for managing root caries among high-risk adults, such as Medicaid beneficiaries with limited access to dental care, remains limited. Studies on the cost-effectiveness of root caries management have mostly targeted children or have not addressed adult Medicaid beneficiaries, who have limited dental care access in the US.^4^ Therefore, the present study’s aims were the evaluation of cost-effectiveness of 38% SDF application in managing high-risk root caries compared with NaF varnish in the context of adult Medicaid beneficiaries in the US.

## METHODS

This study was reported according to the Consolidated Health Economic Evaluation Reporting Standards (CHEERS) (**Supplementary Table S1)**.^5^

### Model and Setting

**Figure 1.**
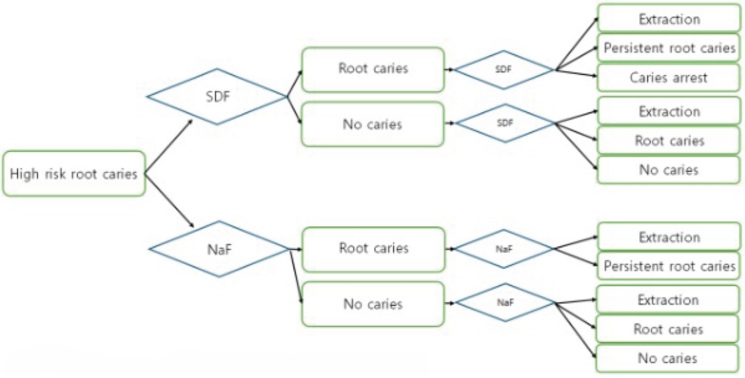
Markov Model for Managing High-Risk Root Caries

A Markov simulation modeling was conducted for the cost-effectiveness analysis of a hypothetical scenario (**Figure 1)**. The study was from a healthcare system perspective under Nevada’s Medicaid fee-for-service program.^6,7^ The model involved a 45-year-old subject with 21 teeth and high risk of root caries in a safety-net ambulatory dental care setting. High risk of root caries was defined as the presence of at least 1 of the following 6 risk factors: (1) diabetes mellitus, (2) substance use disorders including smoking and alcohol, (3) intellectual developmental disabilities, (4) neurodegenerative disorders, (5) mental health conditions, or (6) physical disabilities (**Supplementary Table S2)**. Each tooth could occupy one of the following states:

1. Caries: no clinically documented root caries lesion
2. Root caries: an active root caries lesion documented at the visit
3. Caries arrest: previously active root caries that is clinically assessed as arrested following treatment
4. Persistent root caries: root caries remains active/persists despite treatment
5. Extraction, defined as recorded tooth extraction

Extraction was modeled as an absorbing state, such that once a tooth was extracted, it remained in the extracted state for the rest of the model. Extraction reflects real-world extraction observed in this safety-net setting and is not restricted to root-caries-only etiology.

The time horizon was set at 19 years in the model. This horizon was chosen to align with the adult Medicaid beneficiary age range considered in the Nevada policy context relevant to this study; starting from age 45 years, it captures outcomes through age 64 years. The model was run in annual cycles, with each annual cycle incorporating two 6-month transitions to reflect semiannual preventive visits and treatment decisions. The analysis was conducted at the tooth levels. A subject would begin with initial prevalence of root caries (0.68) and would transition to either with or without caries at the first 6 months. Then, a subject would proceed to choose either SDF application or NaF varnish options at the second 6 months. The first 6-month transition reflected the natural history of disease progression, whereas the second 6-month transition represented the preventive or treatment visit, during which the assigned strategy was applied. During the second 6-month interval, each tooth was managed according to the assigned strategy, either 38% SDF application or 5% NaF varnish. We modeled 38% SDF as both preventive (applied to surfaces without caries to reduce incidence) and therapeutic (applied to active root caries to promote arrest), consistent with its common clinical use in high-risk populations. In contrast, 5% NaF varnish was modeled as preventive only, reflecting its primary role in caries prevention and the limited evidence for its use in lesion arrest in this context. Following SDF and NaF varnish application, the teeth with no caries would either remain without caries, develop caries, or be extracted. Following SDF application, the teeth with root caries would either become arrested, persistent, or extracted. Following NaF varnish, the teeth with root caries would either become persistent or extracted. This prespecified structural distinction is illustrated in **Figure 1**, where the state-specific transition pathways differ by strategy. At the beginning of the subsequent cycle, teeth in the no-caries and caries-arrest states were assigned to the no-caries state, whereas teeth in the root caries and persistent root caries states were assigned to the root caries state. Extraction remained in the extraction state. We set the tooth-years free of extraction as the effect and calculated the incremental cost-effectiveness ratio (ICER) to compare the costs and effects of the SDF application with the situation of NaF varnish. Incremental net monetary benefit (NMB) was calculated as the net difference of monetary benefit by subtracting NaF’s NMB from SDF’s NMB. We used US$50 000 per extraction-free tooth-year as a reference value to facilitate interpretation of the ICER, while recognizing that no established willingness-to-pay (WTP) threshold exists for tooth-level dental outcomes in the Medicaid context.^8^ TreeAge Pro Healthcare 2025 (TreeAge Software) was used for modeling and data analysis.

### Parameters

Transition probabilities and utilities were estimated from tooth-level data obtained from the Nevada Interprofessional Healthy Aging Network (NIHAN), University of Nevada, Las Vegas (UNLV) Health, UNLV Dental Practice, and other published literature (**Supplementary Table S3**).^2,9–17^ UNLV Dental Practice recorded detailed oral health condition data for each tooth in each patient at each visit, which were aggregated to estimate tooth-level transition frequencies and average annual transition probabilities.

Key transition probabilities included root caries incidence among teeth without caries, caries arrest following SDF application, persistence of active root caries after treatment, and extraction. These probabilities were used to inform transitions among the Markov health states. The present study received ethical approval from the UNLV Institutional Review Board (IRB, #1510973-5), under the NIHAN database compiled from records collected at UNLV Health and UNLV Dental Practice.

All costs were expressed in US dollars at the time of the study in 2025. Costs included intervention costs (38% SDF or 5% NaF varnish), oral examination costs, radiography costs, and selected access-related nonmedical costs, including care assistance and transportation. Costs were framed based on charges from UNLV Dental Practice. Cost breakdown for each treatment option with ancillary costs is presented in **Supplementary Table S4**. Out-of-pocket costs for special needs assistance during dental office visits were estimated by aggregating costs of traveling costs between residential addresses and UNLV Dental Practice and labor productivity loss during this traveling time.^18^ Accordingly, the analysis was conducted primarily from a healthcare system perspective, with the additional inclusion of selected nonmedical costs relevant to care access in this high-risk population. A 3% annual discount rate was applied to both costs and effects. The β-distribution was applied for transition probabilities, and the γ-distribution was applied for costs. Model parameters are demonstrated in **Supplementary Table S4**. Demographics, morbidities, and special needs assistance during dental office visit profiles of UNLV Health and Dental Practice subjects were presented in **Supplementary Table S3.**

### Sensitivity Analyses

**One-way sensitivity analysis**: In the one-way sensitivity analysis, we evaluated the impact of the change of one specific parameter value within 95% confidence intervals. Costs of SDF and NaF varnish were set as ranges of $31 to $47 and $27 to $39, respectively. Costs of oral examination, diagnostic imaging, and care assistance/transportation were set as ranges between $27 to $45, $37 to $53, and $93 to $165, respectively. Ranges for one-way sensitivity analyses were based on publicly available fee schedules and charge data from the American Dental Association, the UNLV Dental Practice, and the Nevada Medicaid Dental Services Program; where applicable, ranges were aligned with reported uncertainty (eg, confidence intervals) in the source data. All ranges and sources are summarized in **Supplementary Table S3.**

**Probabilistic sensitivity analysis**: To characterize the uncertainty in the analysis, 1000 Monte Carlo simulations were run with the value of each model input being randomly drawn from the assigned parametric distribution.

## RESULTS

### Base-Case Cost-Effectiveness Analysis

In the base-case scenario (19-year horizon; 21 residual teeth; initial prevalence of root caries among high-risk adults = 0.68), the total discounted cost for the NaF varnish strategy was US$8863, with an effectiveness of 1.39 extraction-free tooth-years. The semiannual 38% SDF strategy had a total discounted cost of US$16 660 and an effectiveness of 7.73 extraction-free tooth-years. Compared with NaF varnish, SDF resulted in an incremental cost of US$7798 and an incremental effectiveness of 6.34 extraction-free tooth-years, yielding an ICER of US$1229 per extraction-free tooth-year gained, which was far below the prespecified WTP threshold of US$50 000. Net monetary benefit was US$60 491 for NaF and US$369 846 for SDF, corresponding to an incremental NMB of US$309 355 (SDF − NaF) (**Table 1**).

**Table 1.** Cost-Effectiveness Analysis Results by 38% SDF and 5% NaF Strategies

**Strategy**	**Cost (US$)**	**Incremental Cost (US$)**	**Effectiveness (Extraction-free Tooth-Year)**	**Incremental Effectiveness (Extraction-free Tooth-Year)**	**ICER**	**NMB**
NaF	8863		1.39			60 491
SDF	16 660	7798	7.73	6.34	1229	369 846

Abbreviations: ICER, incremental cost-effectiveness ratio; NaF, sodium fluoride varnish; NMB, net monetary benefit; SDF, sodium diamine fluoride.

### One-Way Sensitivity Analyses

Across all one-way sensitivity analyses, the ICERs for SDF remained well below the WTP threshold, indicating robust cost-effectiveness (**Table 2**). Overall, the base-case findings and one-way sensitivity analyses consistently supported that semiannual 38% SDF is a cost-effective strategy compared with 5% NaF varnish under the specified WTP threshold.

**Table 2.** One-Way Sensitivity Analysis Results

**Parameter Varied**	**Range Tested (US$)**	**ICER**	**Interpretation**
SDF cost	31	1164	Higher SDF cost increased ICER; conclusion unchanged
	39	1229	
	47	1295	
NaF cost	27	1235	ICER changed minimally; SDF remained cost-effective across range
	33	1228	
	39	1222	
Care assistance/transportation cost	93	1054	Largest ICER swing among tested parameters; SDF was still cost-effective
	129	1240	
	165	1425	
Oral examination cost	27	1188	Moderate ICER change; conclusion unchanged
	36	1234	
	45	1281	
Radiography cost	37	1218	Small ICER change; conclusion unchanged
	45	1231	
	53	1244	

Abbreviations: ICER, incremental cost-effectiveness ratio; NaF, sodium fluoride varnish; SDF, sodium diamine fluoride.

### Probabilistic Sensitivity Analysis

**Figure 2.**
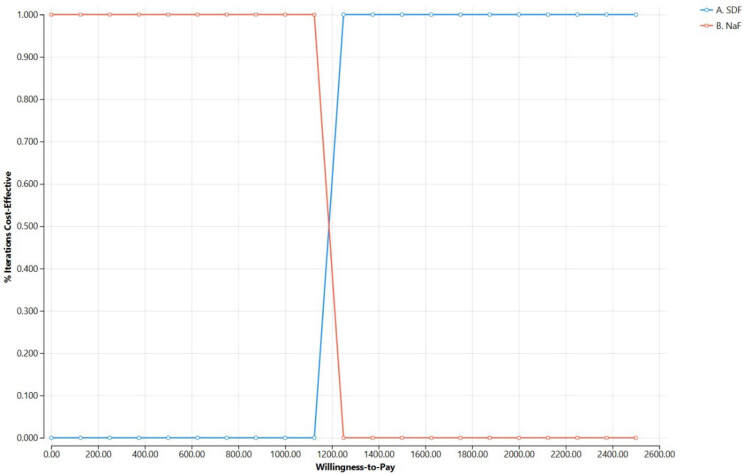
Cost-Effectiveness Acceptability Curve, as Probabilistic Sensitivity Analysis Results Abbreviations: ICER, incremental cost-effectiveness ratio; NaF, sodium fluoride varnish; SDF, sodium diamine fluoride. The x-axis shows willingness-to-pay (WTP) per utility-weighted tooth-year gained, and the y-axis shows the proportion of PSA iterations in which each strategy is cost-effective. At very low WTP values, NaF varnish is cost-effective in nearly all iterations, whereas 38% SDF becomes cost-effective in nearly all iterations once WTP exceeds approximately US$1229, consistent with the base-case ICER.

Tost-effectiveness acceptability curves are shown in **Figure 2**. At very low WTP values, NaF varnish is cost-effective in nearly all iterations, whereas 38% SDF becomes cost-effective in nearly all iterations once WTP exceeds approximately US$1229, consistent with the base-case ICER.

### Variation in Fluoride Intervention Costs

When the SDF unit cost varied from US$31 to US$47, the ICER ranged from US$1164 to US$1295 per extraction-free tooth-year gain. When the NaF varnish unit cost varied from US$27 to US$39, the ICER ranged from US$1235 to US$222 per extraction-free tooth-year gain.

## DISCUSSION

The present study evaluated the cost-effectiveness of SDF application in managing high-risk root caries among adult Medicaid beneficiaries, an area with limited economic evidence for adult high-risk populations in safety-net settings. A healthcare system perspective was applied at safety-net dental ambulatory care settings. SDF application proved to be more effective than NaF varnish at managing high-risk root caries. Compared with 5% NaF varnish, semiannual 38% SDF application was cost effective for managing high-risk root caries with a difference of 6.34 extraction-free tooth-years and an additional cost of $7798. The ICER was $1229 per extraction-free tooth-year gained and incremental NMB was $309 355.

These findings imply that semiannual 38% SDF application might reduce and delay tooth extraction due to root caries. These findings underscore the insights for the adult Medicaid beneficiaries who have limited dental care access because certain states, including Nevada, cover only emergency extractions, palliative care, and pregnancy-related dental services.^17,19^ SDF can be reapplied at an affordable cost at institutional care settings (eg, long-term care and correctional facilities). The cost-effectiveness benefits of SDF application can be disseminated even by non-dental providers (eg, primary care providers) in statewide dental provider shortage areas, particularly “dental deserts” where Nevada Medicaid-accepting dentists are not available in the countywide radius.^17^ Primary care providers may share oral health services with dental providers in the alignment of teledentisty.^17^ Care models that incorporate teledentistry and interprofessional collaboration may help expand preventive and caries-arrest services while maintaining referral pathways for definitive dental treatment. An integrated orosystemic care model is underscored to train the healthcare workforce to disseminate the benefits of SDF application for managing root caries.

In contrast to prior pediatric economic evaluations of SDF that was modeled for school- or community-based prevention programs and may underestimate caregiver time costs, our analysis was grounded in a Medicaid safety-net delivery context.^15^ Specifically, we adopted a healthcare system perspective under Nevada Medicaid fee-for-service and incorporated cost components that are particularly salient for high-risk adults, including special needs–related assistance and transportation/time costs associated with dental visits. By reflecting these access-related resource requirements, the model better represents the real-world economic consequences of delivering preventive and caries-arrest services to medically and socially complex adult Medicaid populations.

This distinction also strengthens the policy relevance of our findings. Whereas pediatric studies typically inform the expansion of school- or community-based prevention programs, our results speak to implementation in dental provider shortage areas, where timely access to Medicaid-accepting dentists is limited and noninvasive interventions may be deployed to reduce disease progression and delay extractions. The findings are particularly timely as Medicaid dental financing and delivery increasingly transition from fee-for-service toward managed care models that emphasize efficiency and scalable care pathways.^19,20^

In the US Medicaid context, WTP thresholds for dental outcomes are not well established. Therefore, we used US$50 000 per extraction-free tooth-year only as a heuristic reference to aid interpretation and comparison with commonly used benchmarks in economic evaluation, rather than as a formal decision threshold. Because the model generated outcomes at the tooth level, we additionally considered a more pragmatic policy-relevant reference value of approximately US$2500 per extraction-free tooth-year, which corresponds to distributing US$50 000 across approximately 20 teeth. Notably, this magnitude is consistent with the annual cap of approximately US$2500 per beneficiary observed in Nevada dental benefit expansions for certain adult populations.^21,22^ Regardless of whether the reference point was US$50 000 or the pragmatic tooth-level benchmark, the ICER remained far below these values, supporting the cost-effectiveness of semiannual 38% SDF application.

The present study had several assumptions and limitations. Real-world extraction rates in this study were higher than in previous literature. We speculate that the some of the extractions might have occurred for reasons other than root caries. Extractions were assumed not to be only from root caries but from real-world data that reflected higher rates than previously reported.^11,12,14,15^ In addition, the indirect cost resulting from special needs assistance during dental office visits was estimated and applied based on real-world safety-net practice, where dental services were provided for those with more complex health conditions under Medicaid than private practice.^9,13^ The model was simplified and did not model the other dental care options (eg, root canal treatment that was included in the model elsewhere).^15^ The data source was limited to a single Medicaid-accepting public practice in a single geographic location. The transition probability estimates were derived from tooth-level clinical records from UNLV Dental Practice, in which tooth status was recorded at each visit and aggregated to estimate transition frequencies across model states. This limitation might not represent the analysis results across the type of payor and practice patterns even in the same geographic location. Accordingly, the present estimates primarily reflect care delivery in a Nevada safety-net Medicaid-accepting clinic, and their generalizability to other states or settings may vary depending on Medicaid benefit design, clinical practice patterns, and population case mix. Our findings should be interpreted in light of the underlying data source, model structure, and analytic assumptions. Nevertheless, the findings of the present study may be particularly relevant to states such as Arizona, Florida, and Texas, which have dental service profiles for adult Medicaid beneficiaries aged 21 to 64 years, similar to that of Nevada.^21^ Finally, in the US healthcare system, particularly in state Medicaid payment systems, no predetermined WTP threshold for dental care services has been established. Accordingly, future work should combine threshold and budget impact analyses with stakeholder engagement to develop policy-relevant value benchmarks, using existing benefit caps (eg, Nevada’s ~US$2500 annual cap) as contextual reference points.^21,22^ Further price-threshold analysis and Medicaid Reinvestment Advisory Committee’s involvement in determining WTP for dental care services might be next steps to establish WTP consensus.

## CONCLUSIONS

Semiannual 38% SDF was cost-effective vs 5% NaF varnish for managing high-risk root caries among adult Medicaid beneficiaries under Nevada’s Medicaid fee-for-service plan. SDF increased effectiveness by 6.34 extraction-free tooth-years at an additional cost of US$7798 (ICER: US$1229 per extraction-free tooth-year), and results were robust in sensitivity analyses. Within the assumptions of this tooth-level model and the single-site Nevada safety-net data source, semiannual SDF may reduce and delay tooth extraction in dental provider shortage areas, warranting validation with multi-site data and policy-relevant implementation studies.

### Institutional Review Board Statement

The present study received ethical approval from University of Nevada, Las Vegas (UNLV) Institutional Review Board (IRB, #1510973-5) on May 5, 2023.

## Supplementary Material

Online Supplementary Material
