# Supplementary material for: Managing High-Risk Root Caries with Silver Diamine Fluoride Application: A Markov Simulation Study
Source: J Health Econ Outcomes Res. 2026 May 14;13(1):168–73. doi: 10.36469/001c.160210 (PMC13179781; doi:10.36469/001c.160210)
Supplement: Online Supplementary Material [file jheor_2026_13_1_160210_344594.pdf]

Journal of Health Economics and Outcomes Research

Online Supplementary Material

Managing High-Risk Root Caries with Silver Diamine Fluoride Application: A Markov Simulation Study.
JHEOR. 2026;13(1):168-173. doi:10.36469/jheor.2026.160210

Table S1: CHEERS 2022 Checklist

Table S2: Demographics, Morbidities and Special Needs Assistance Profiles of UNLV Health and Dental Practice Subjects

Table S3: Model Parameters

Table S4: Breakdown Costs of Choosing 38% SDF Application and 5% NaF Varnish Options and Ancillary Costs (US$)

This supplementary material has been provided by the authors to give readers additional information about their work.

This is an open-access article distributed under the terms of the Creative Commons Attribution 4.0 International License (CCBY-4.0). View this license's legal deed at http://creativecommons.org/licenses/by/4.0 and legal code at http://creativecommons.org/licenses/by/4.0/legalcode for more information.

Table S1. CHEERS 2022 Checklist

Topic	No.	Item	Location
Title	1	Identify the study as an economic evaluation and specify the interventions being compared.	Title, Page 1
Abstract	2	Provide a structured summary that highlights context, key methods, results, and alternative analyses.	Abstract, Page 1
Introduction			
Background and objectives	3	Give the context for the study, the study question, and its practical relevance for decision making in policy or practice.	Introduction, Lines 55 to 79
Methods			
Health economic analysis plan	4	Indicate whether a health economic analysis plan was developed and where available.	Methods, Lines 83 to 84
Study population	5	Describe characteristics of the study population (such as age range, demographics, socioeconomic, or clinical characteristics).	Methods, Lines 88 to 95, **Table S2**
Setting and location	6	Provide relevant contextual information that may influence findings.	Methods, Lines 88 to 89
Comparators	7	Describe the interventions or strategies being compared and why chosen.	Methods, Lines 96 to 102
Perspective	8	State the perspective(s) adopted by the study and why chosen.	Methods, Lines 80 to 81
Time horizon	9	State the time horizon for the study and why appropriate.	Methods, Lines 88 to 89
Discount rate	10	Report the discount rate(s) and reason chosen.	Methods, Lines 133 to 134
Selection of outcomes	11	Describe what outcomes were used as the measure(s) of benefit(s) and harm(s).	Methods, Lines 123 to 124
Measurement of outcomes	12	Describe how outcomes used to capture benefit(s) and harm(s) were measured.	Methods, Lines 107 to 108
Valuation of outcomes	13	Describe the population and methods used to measure and value outcomes.	Methods, Lines 108 to 112, and **Table S3**
Measurement and valuation of resources and costs	14	Describe how costs were valued.	Methods, Lines 128 to 136 and **Table S4**
Currency, price date, and conversion	15	Report the dates of the estimated resource quantities and unit costs, plus the currency and year of conversion.	Methods, Lines 128 to 136 and **Table S4**
Rationale and description of model	16	If modelling is used, describe in detail and why used. Report if the model is publicly available and where it can be accessed.	Methods, Lines 88 to 89, **Figure 1**; Lines 313 to 314, data availability statement
Analytics and assumptions	17	Describe any methods for analysing or statistically transforming data, any extrapolation methods, and approaches for validating any model used.	Methods, Lines 134 to 135
Characterising heterogeneity	18	Describe any methods used for estimating how the results of the study vary for subgroups.	Methods, Lines 142 to 152, one-way sensitivity analysis
Characterising distributional effects	19	Describe how impacts are distributed across different individuals or adjustments made to reflect priority populations.	Methods, Lines 142 to 152, one-way sensitivity analysis
Characterising uncertainty	20	Describe methods to characterise any sources of uncertainty in the analysis.	Methods, Lines 154 to 158, probabilistic sensitivity analysis
Approach to engagement with patients and others affected by the study	21	Describe any approaches to engage patients or service recipients, the general public, communities, or stakeholders (such as clinicians or payers) in the design of the study.	Not applicable

Table S1. CHEERS 2022 Checklist

Topic	No.	Item	Location
Results			
Study parameters	22	Report all analytic inputs (such as values, ranges, references) including uncertainty or distributional assumptions.	Results, Lines 160 to 171, **Table 1**, base-case cost-effectiveness analysis
Summary of main results	23	Report the mean values for the main categories of costs and outcomes of interest and summarise them in the most appropriate overall measure.	Results, Lines 160 to 171, **Table S3**
Effect of uncertainty	24	Describe how uncertainty about analytic judgments, inputs, or projections affect findings. Report the effect of choice of discount rate and time horizon, if applicable.	Results, Lines 175 to 192, **Table 2** and **Figure 2**
Effect of engagement with patients and others affected by the study	25	Report on any difference patient/service recipient, general public, community, or stakeholder involvement made to the approach or findings of the study	Not applicable
Discussion			
Study findings, limitations, generalisability, and current knowledge	26	Report key findings, limitations, ethical or equity considerations not captured, and how these could affect patients, policy, or practice.	Discussion
Other relevant information			
Source of funding	27	Describe how the study was funded and any role of the funder in the identification, design, conduct, and reporting of the analysis	End of manuscript (funding resources)
Conflicts of interest	28	Report authors conflicts of interest according to journal or International Committee of Medical Journal Editors requirements.	End of manuscript (conflict of interest statement)

REFERENCE

Husereau D, Drummond M, Augustovski F, et al. Consolidated Health Economic Evaluation Reporting Standards 2022 (CHEERS 2022) Explanation and elaboration: a report of the ISPOR CHEERS II Good Practices Task Force. *Value Health*. 2022;25. doi:10.1016/j.jval.2021.10.008

Table S2. Demographics, Morbidities and Special Needs Assistance Profiles of UNLV Health and Dental Practice Subjects

**Variable**	**N = 1703**
Age, range (mean ± SD)	21-64 (47.2 ± 11.9)
Sex, % (n)	
Male	40.9 (696)
Female	59.1 (1007)
Race/ethnicity, % (n)	
White	54.4 (926)
Black	12.9 (220)
Hispanic	16.1 (273)
Asian/Pacific Islander	7.4 (127)
Mixed and other	9.2 (157)
Comorbidities, % (n)	
Diabetes mellitus	43.7 (744)
Substance use disorders: Smoking, alcohol, opioid, cannabis, cocaine, other substances	47.0 (800)
Intellectual developmental disabilities and degenerative neurologic disorders	23.0 (392)
Mental health conditions: Mood disorders, schizophrenia, other non-mood psychotic disorders, anxiety, and stress-related somatoform disorders	52.1 (886)
Physical disabilities: Cord injuries, extremities amputation, and limited gait and balance requiring ambulatory device use	12.4 (211)
Special needs assistance during dental office visit	42.7 (728)

Table S3. Model Parameters

**Parameters**	**Value**	**95% Range**	**References**
Baseline No. of residual teeth	21	18-26	UNLV Health, UNLV Dental Practice, GRS 12th ed., Mitchell et al
Initial prevalence of root caries among high risk	0.68	0.54-0.81	UNLV Health, UNLV Dental Practice, Zaffarano et al
Probability from caries to extraction with SDF	0.09	0.02-0.06	Zaffrano et al, Chan et al, Li et al, GRS 12th ed., UNLV Dental Practice
Probability of persistent root caries with SDF	0.51	0.46-0.65	Zaffrano et al, Chan et al, Li et al, Kettlekamp et al, UNLV Dental Practice
Probability of caries arrest with SDF	0.40	0.33-0.46	Chan et al, Kettlekamp et al, UNLV Dental Practice
Probability from no carries to extraction with SDF	0.05	0.02-0.07	Zaffrano et al, Chan et al, UNLV Dental Practice
Probability from no carries to caries with SDF	0.29	0.27-0.53	Zaffrano et al, Chan et al, GRS 12th ed., UNLV Dental Practice
Probability of no carries with SDF	0.66	0.44-0.69	Zaffrano et al, Chan et al, Li et al, UNLV Dental Practice
Probability from caries to extraction with NaF varnish	0.35	0.29-0.42	GRS 12th ed., UNLV Dental Practice
Probability of persistent caries with NaF varnish	0.65	0.57-0.74	Zhu et al, UNLV Dental Practice
Probability from no carries to extraction with NaF varnish	0.19	0.14-0.23	Zhu et al, UNLV Dental Practice
Probability from no carries to root caries with NaF varnish	0.49	0.43-0.57	Zhu et al, UNLV Dental Practice
Probability of no carries with NaF varnish	0.32	0.28-0.37	Zhu et al, UNLV Dental Practice
Utility of arrest	0.81	0.67-0.92	UNLV Dental Practice, Li et al, Zhu et al
Utility of caries	0.47	0.40-0.55	UNLV Dental Practice, Li et al, Zhu et al
Utility of extraction	0.10	0.00-0.20	UNLV Dental Practice, Li et al, Zhu et al
Cost (US $)			
SDF application	39	31-47	American Dental Association. UNLV Dental Practice, Nevada Medicaid Dental Services Program

Table S3. Model Parameters

Parameters	Value	95% Range	References
NaF varnish	32	27-39	American Dental Association. UNLV Dental Practice, Nevada Medicaid Dental Services Program
Care assistance and transportation	127	93-165	American Dental Association. UNLV Dental Practice, Nevada Medicaid Dental Services Program
Oral examination	35	27-45	American Dental Association. UNLV Dental Practice, Nevada Medicaid Dental Services Program
Diagnostic imaging	44	37-53	American Dental Association. UNLV Dental Practice, Nevada Medicaid Dental Services Program
Caries risk assessment and documentation	30	24-40	American Dental Association. UNLV Dental Practice, Nevada Medicaid Dental Services Program
Extraction	271	226-349	American Dental Association. UNLV Dental Practice, Nevada Medicaid Dental Services Program

REFERENCES

1. NIHAN and UNLV Health. Chart review. Nevada Interprofessional Healthy Aging Network; 2025.
2. UNLV Dental Practice. Chart review. University of Nevada, Las Vegas; 2025.
3. American Geriatrics Society. *Multicoplexity – Geriatric Syndrome: Dentistry and Oral Health.* 12th ed. New York, NY: American Geriatrics Society; 2025. ISBN 1-886775-68-0.
4. Mitchell C, Gross AJ, Milgrom P, Mancl L, Prince DB. Silver diamine fluoride treatment of active root caries lesions in older adults: a case series. *J Dent.* 2021;106:103561. doi:10.1016/j.jdent.2020.103561
5. Zaffarano L, Salerno C, Campus G, et al. Silver diamine fluoride (SDF) efficacy in arresting cavita caries lesions in primary molars: A systematic review and metaanalysis. *Int J Environ Res Public Health*. 2022;19:12917. doi:10.3390/ijerph191912917
6. Chan AYK, Chu S, Yu OY, Chu CH. Clinical use of silver diamine fluoride in older adults: a scoping review. *J Dent*. 2025;162:106019. doi:10.1016/j.jdent.2025.106019
7. Li R, Lo EC, Liu BY, Liu MC, Chu CH. Randomized clinical trial on arresting dental caries through silver diamine fluoride applications in community-dwelling elders. *J Dent*. 2016;51:15-20. doi:10.1016/j.jdent.2016.05.005
8. Kettlekamp K, Desai J, Lewis S, Comnick C, Marchini L. Outcomes of silver diamine fluoride treatment among older adults: a retrospective analysis. *Spec Care Dent*. 2025;45:e70013. doi:10.1111/scd.70013
9. Zhu LW, Wang RX, Zhang Y, Zhan JY, Lu HX, Chen X. A cost-effectiveness analysis of fluoride varnish application in preventing root caries in elderly persons: a Markov simulation study. *BMC Oral Health*. 2024;24:483. doi:10.1186/s12903-024-04226-5.
10. Medicaid fee schedules. American Dental Association. Accessed November 25, 2025. https://www.ada.org/advocacy/advocacy-issues/medicaid/medicaid-fee-schedules
11. Nevada Medicaid. Search fee schedule; Dental services program. Nevada Health Authority. Accessed November 25, 2025. https://www.medicaid.nv.gov/hcp/provider/Resources/SearchFeeSchedule/tabid/528/Default.aspx
12. Nevada Medicaid. Provider type 22 billing guide and fee schedules; Dental services. Nevada Health Authority. Accessed November 25, 2025. https://www.medicaid.nv.gov/Downloads/provider/NV_BillingGuidelines_PT22.pdf

Table S4. Breakdown Costs of Choosing 38% SDF Application and 5% NaF Varnish Options and Ancillary Costs (US$)

Options	Transitions		Costs US$		
			First 6 Mo (A)	Second 6 Mo (B)	Total Annual (A+B)
38% SDF	Root caries	Extraction	275	472	747
		Persistent root caries	275	231	506
		Carries arrest	275	201	476
	No caries	Extraction	245	472	717
		Root caries	245	231	476
		No caries	245	201	446
NaF varnish	Root caries	Extraction	268	465	733
		Persistent root caries	268	224	492
	No caries	Extraction	238	465	703
		Root caries	238	224	462
		No caries	238	194	432

Abbreviations: NaF, sodium fluoride; SDF, silver diamine fluoride.
